# EurOP^2^E – the European Open Platform for Prescribing Education, a consensus study among clinical pharmacology and therapeutics teachers

**DOI:** 10.1007/s00228-021-03101-4

**Published:** 2021-02-23

**Authors:** Michiel J. Bakkum, Milan C. Richir, Paraskevi Papaioannidou, Robert Likic, Emilio J. Sanz, Thierry Christiaens, João N. Costa, Romaldas Mačiulaitis, Lorena Dima, Jamie Coleman, Jelle Tichelaar, Michiel A. van Agtmael, for the Education Working Group of the European Association for Clinical Pharmacology and Therapeutics (EACPT) and its affiliated Network of Teachers in Pharmacotherapy (NOTIP), Ivanka Atanasova, Maria Ganeva, Emil Gatchev, I.I. Kostadinova, S. Mimica Matanovic, D. Vitezic, Greta Wozniak, E. Kmonickova, Karel Urbanek, P. Damkier, R. K. Huupponen, Marine Auffret, T. Bejan-Angoulvant, Laurent Chouchana, Jean-Luc Cracowski, M. D. Drici, J. L. Faillie, Hélène Geniaux, M. Molimard, D. Orlikowski, Karine Palin, Y-M Pers, Nicolas Picard, N. Simon, E. Toussirot, R. H. Boger, I. Cascorbi, S. C. Mueller, R. Regenthal, M. Schwab, M. S. Schwaninger, P. A. Thuermann, L. Wojnowski, D. Kouvelas, P. Riba, David M. Kerins, David J. Williams, M. Cosentino, Fabrizio De Ponti, Amelia Filippelli, R. Leone, Vittorio Locatelli, Baiba Jansone, Romaldas Gulbinovic, Janet Mifsud, Jan J. Braszko, I. Kocic, Luiza Breitenfeld, M. Castelo-Branco, Simona Conea, Ioan Magyar, S. Bevc, Mojca Krzan, M. L. Bernal, D. Capellà, A. Carcas, F. J. De Abajo, M. Lopez-Rico, M. I. Lucena, C. Pontes, E. J. Sanz, Y. Böttiger, Madeleine Le Grevès, I. de Waard-Siebinga, Ben J. A. Janssen, Wilma Knol, Rahul Pandit, F. van Rosse, G. Dent, Albert Ferro, A. W. Hitchings, V. Kapil, K. D. Linton, Y. K. Loke, Michael Okorie, Richard David Plumb, Sarah Pontefract, S. Ranmuthu, A. P. Sampson, H. K. R. Thanacoody, Jonathan P. Whitfield, Kurt Wilson

**Affiliations:** 1 Department of Internal Medicine, Section Pharmacotherapy, Amsterdam UMC, Vrije Universiteit Amsterdam, De Boelelaan 1117, 1081 Amsterdam, HV Netherlands; 2 Research and Expertise Centre in Pharmacotherapy Education (RECIPE), De Boelelaan 1117, 1081 HV Amsterdam, The Netherlands; 3 European Association for Clinical Pharmacology and Therapeutics (EACPT) Education Working Group, Athens, Greece; 4 Faculty of Health Sciences, School of Medicine, Department of Pharmacology, Aristotle University of Thessaloniki, Thessaloniki, Greece; 5 Unit of Clinical Pharmacology, University of Zagreb School of Medicine and Clinical Hospital Centre Zagreb, Zagreb, Croatia; 6 School of Health Sciences, Universidad de La Laguna, Tenerife, Spain; 7 Section Clinical Pharmacology, Heymans Institute of Pharmacology Ghent University, Ghent, Belgium; 8 Department of Pharmacology and Clinical Pharmacology, University of Lisbon, Lisbon, Portugal; 9 Lithuanian University of Health Sciences, Institute of Physiology and Pharmacology, Kaunas, Lithuania; 10 Faculty of Medicine, Transilvania University of Brașov, Brașov, Romania; 11 University of Birmingham, Institute of Clinical Sciences, Birmingham, UK

**Keywords:** Open educational resources, Digital education, Clinical pharmacology and therapeutics, Medical education

## Abstract

**Purpose:**

Sharing and developing digital educational resources and open educational resources has been proposed as a way to harmonize and improve clinical pharmacology and therapeutics (CPT) education in European medical schools. Previous research, however, has shown that there are barriers to the adoption and implementation of open educational resources. The aim of this study was to determine perceived opportunities and barriers to the use and creation of open educational resources among European CPT teachers and possible solutions for these barriers.

**Methods:**

CPT teachers of British and EU medical schools completed an online survey. Opportunities and challenges were identified by thematic analyses and subsequently discussed in an international consensus meeting.

**Results:**

Data from 99 CPT teachers from 95 medical schools were analysed. Thirty teachers (30.3%) shared or collaboratively produced digital educational resources. All teachers foresaw opportunities in the more active use of open educational resources, including improving the quality of their teaching. The challenges reported were language barriers, local differences, lack of time, technological issues, difficulties with quality management, and copyright restrictions. Practical solutions for these challenges were discussed and include a peer review system, clear indexing, and use of copyright licenses that permit adaptation of resources.

**Conclusion:**

Key challenges to making greater use of CPT open educational resources are a limited applicability of such resources due to language and local differences and quality concerns. These challenges may be resolved by relatively simple measures, such as allowing adaptation and translation of resources and a peer review system.

**Supplementary Information:**

The online version contains supplementary material available at 10.1007/s00228-021-03101-4.

## Introduction

Clinical pharmacology and therapeutics (CPT) education throughout Europe insufficiently prepares medical students and young doctors to prescribe safely [1]. There are large differences in the quantity and quality of CPT education in different European medical schools. Furthermore, the majority of European CPT curricula use a traditional rather than problem-based teaching style [2]; even though the latter is more appropriate because prescribing is a complex skill that requires knowledge, skills, attitudes, and active training [3]. Previous research has shown that students who had a problem-based education significantly outperformed their traditionally educated peers in a case-based examination [1, 4]. The Education Working Group of the European Association for Clinical Pharmacology and Therapeutics (EACPT) proposes to improve education and training in prescribing knowledge and skills, by harmonizing medical curricula and making them more problem-based [2]. For this purpose, several initiatives, such as the establishment of universal learning outcomes for undergraduate CPT [5] and the introduction of a pan-European prescribing skills examination [6], have been introduced. Another promising strategy to achieve harmonization is to create educational resources collaboratively and to actively share existing resources. To this end, the EACPT education working group aims to launch an online platform for open educational resources and collaboration amongst teachers.

Open educational resources are typically digital learning and teaching materials that are freely accessible that can be revised and redistributed by anyone other than the original author [7]. In addition to their potential to augment and harmonize international education, without forgoing the need for local adaptation, these resources may reduce educational costs, increase accessibility, and promote continual improvement. However, the implementation of open educational resources in existing curricula is challenging [8–13]. The “not invented here syndrome”, questionable quality, and a lack of time to find appropriate open educational resources and/or adapting them are previously reported examples of barriers to the use of these resources mentioned by teachers. These and other potential barriers (such as national differences in CPT curricula, prescribing guidelines, and drug availability) challenge to the uptake of our proposed collective online platform. Therefore, the aims of this study were to determine opportunities and challenges perceived by European CPT teachers to creating and implementing open educational resources and identifying solutions to potential challenges.

## Methods

First an online survey was done about teachers’ current use of, and opinion about, digital and open educational resources. Secondly an international consensus meeting was held at the 2019 EACPT conference in Stockholm, Sweden.

### Part 1: The online survey

An invitation to participate in the survey was sent by email to all known CPT teachers of the medical schools in all 28 EU states (including the UK, prior to Brexit). The single medical school in Luxembourg was later excluded because it did not offer a complete medical degree. The survey was created, and data were collected using the electronic case-report system CastorEDC (www.castoredc.com). The survey was open from 27 March 2019 to 13 May 2019. The questions were divided into four themes: expectations regarding digital education compared to face-to-face teaching, current sharing and collaborating practices, conditions for sharing, and foreseeable advantages and challenges of the online platform. A combination of yes/no, multiple-answer, Likert-type, and open questions was used. The first set of Likert-type questions was adapted from Kirkpatrick’s pyramid of educational outcomes [14]; the original second tier of the pyramid (“learning”) was subdivided into learning of knowledge, skills, and attitudes. A 5-point scale from much worse than face-face teaching [1] to much better than face-to-face education [5] was used. For the second set of Likert-type questions, a 5-point scale with level of agreement ([1] completely disagree to [5] completely agree) was used. The teachers were also asked to describe their current best practice digital educational resources. An overview and characterization of these resources has been published elsewhere [15].

### Part 2: The consensus meeting

The results of the survey, in particular the challenges for the platform, were discussed in a dedicated meeting during the EACPT conference 2019 in Stockholm. Affiliates to the EACPT education working group and its Network Of Teachers In Pharmacotherapy Education (NOTIP) received an invitation beforehand, but the meeting was open to all conference attendees. All challenges and potential solutions were discussed, with participants being asked to suggest additional solutions. Then a plenary discussion was held to reach consensus on which solutions to adopt for the platform. The meeting was audio-recorded, subsequently transcribed verbatim, and is summarized here.

### Copyright license terminology

The Creative Commons (www.creativecommons.org) terminology for copyright licenses was used. There are seven licenses (Table 1*)*, which are modular and contain up to four rights: “By” for Attribution, “SA” for Share-Alike, “NC” for Non-Commercial, and “ND” for No Derivatives, as explained in more detail by Bissel [16]. The first five statements about conditions under which teachers would be willing to share their resources were laymen descriptions of these four rights. The copyright licenses that the teachers felt most comfortable with were determined by combining their answers to these five statements. Neutral answers were considered to agree with the most open variant of copyright (e.g. if someone felt neutral about the need for attribution, this was scored as attribution not required; if someone felt neutral about commercial use, this was scored as commercial use allowed). Educational resources are only considered open if they can be re-used, revised, and redistributed without cost. Therefore, resources with a ND-license are not considered open [16, 17].

**Table 1:**
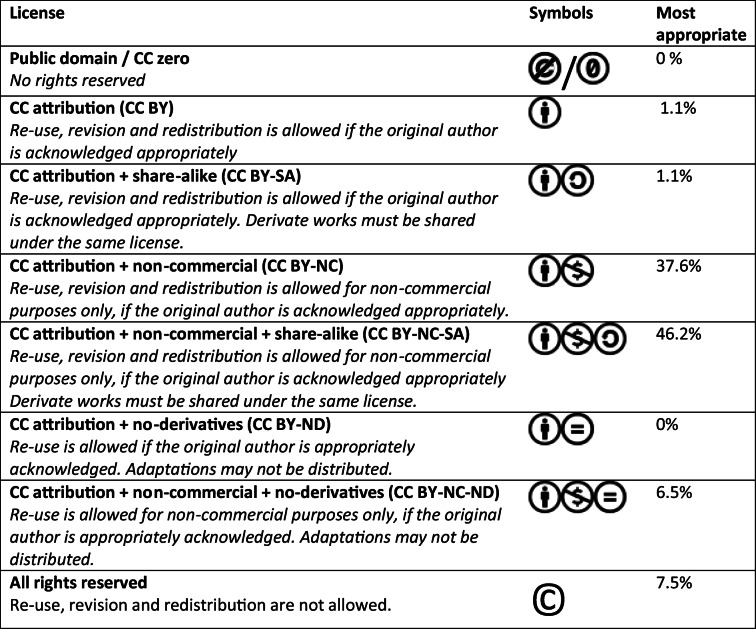
Appropriateness of copyright licenses

The results are based on the combination of individual answers on each of the laymen descriptions of Creative Commons rights (Fig. 2). *CC* Creative Commons, *BY* attribution, *SA* share-alike, *NC* non-commercial, *ND* no derivatives

### Ethical considerations

All participants were required to give their informed consent before the survey. The local ethics committee at Amsterdam UMC, VUmc, declared that the study did not fall within the scope of the Medical Research Involving Human Subjects Act (WMO). The Ethical Review Board of the Dutch Society for Medical Education (NVMO) approved the study protocol (NVMO-ERB 2018.8.12). The intention to record the meeting and use it for research purposes was stated at the beginning of the consensus meeting, and attendees were given the opportunity to leave if they objected. All comments made by non-authors during the meeting were anonymized in the transcript.

### Data analyses

Data were gathered in CastorEDC, downloaded to Microsoft Excel, and analysed using SPSS (IBM version 26.0). The answers to yes/no, multiple-answer, and Likert-type questions are expressed as a percentage (number of participants that chose that answer option relative to the total number of participants). The open-answer questions were analysed using thematic analyses. The choice for a thematic approach was made because it best describes the answer to the “what”-type questions that were asked and because we expected the answers to be insufficiently detailed for a phenomenological approach. Author M.B. categorized the answers in proposed themes and subthemes. All of these themes were subsequently discussed with author J.T. until both authors reached consensus on the final definition and wording. A member check of the final themes was performed by presenting them during the consensus meeting and allowing the participants to question, add to, or propose alterations to these themes.

## Results

Three-hundred ninety-three teachers were invited to participate in this study, 99 of whom answered at least one survey question. They represent 95 (34%) distinct medical schools in the UK and EU countries except Austria and Luxembourg. Teacher demographics and the use of digital resources in medical schools have been reported elsewhere [15].

### Current sharing and collaborating practices

Teachers from 66 of the 95 medical schools (69%) used digital educational resources in their CPT curriculum. Almost half of them (30) indicated that (for at least one of the resources) they shared or collaborated with another institution and half of them (33) did not share or collaborate. In an open-ended question, teachers were asked why they did or did not share/collaborate. The results are presented in Supplementary Table 1*.*

### Opinion and expectations of digital teaching

**Fig. 1:**
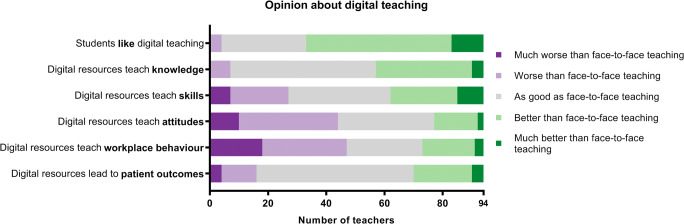
Opinions about digital education compared to face-to-face education. Statements are based on Kirkpatrick’s pyramid model of educational outcomes. Tier 2 (“learning”) was split into knowledge, skills, and attitudes

Ninety-four teachers completed these Likert-type questions. Almost two-thirds (64.9%) believed that students like digital education better (or much better) than face-to-face education. For knowledge acquisition, 92.6% felt that digital education is as effective or more effective than face-to-face teaching, but 46.8% believed digital education to be worse (or much worse) for teaching attitudes. In general, teachers believed that the lower levels (“reaction” and “learning of knowledge, skills, and attitudes”) of the adapted Kirkpatrick pyramid can be effectively taught digitally, but that higher tiers (“behaviour” and “clinical results”) require face-to-face teaching (Fig. 1). With regard to the advantages of digital teaching, the most common answers were the re-usability of digital resources (73 times), the belief that students prefer it (58 times), and possibilities to share the resources with others (54 times). Disadvantages were the time (79 times), costs (65 times), and skills (30 times) required to develop digital educational resources. Supplementary Table 2 shows all advantages and disadvantages.

### Conditions for sharing resources

**Fig. 2:**
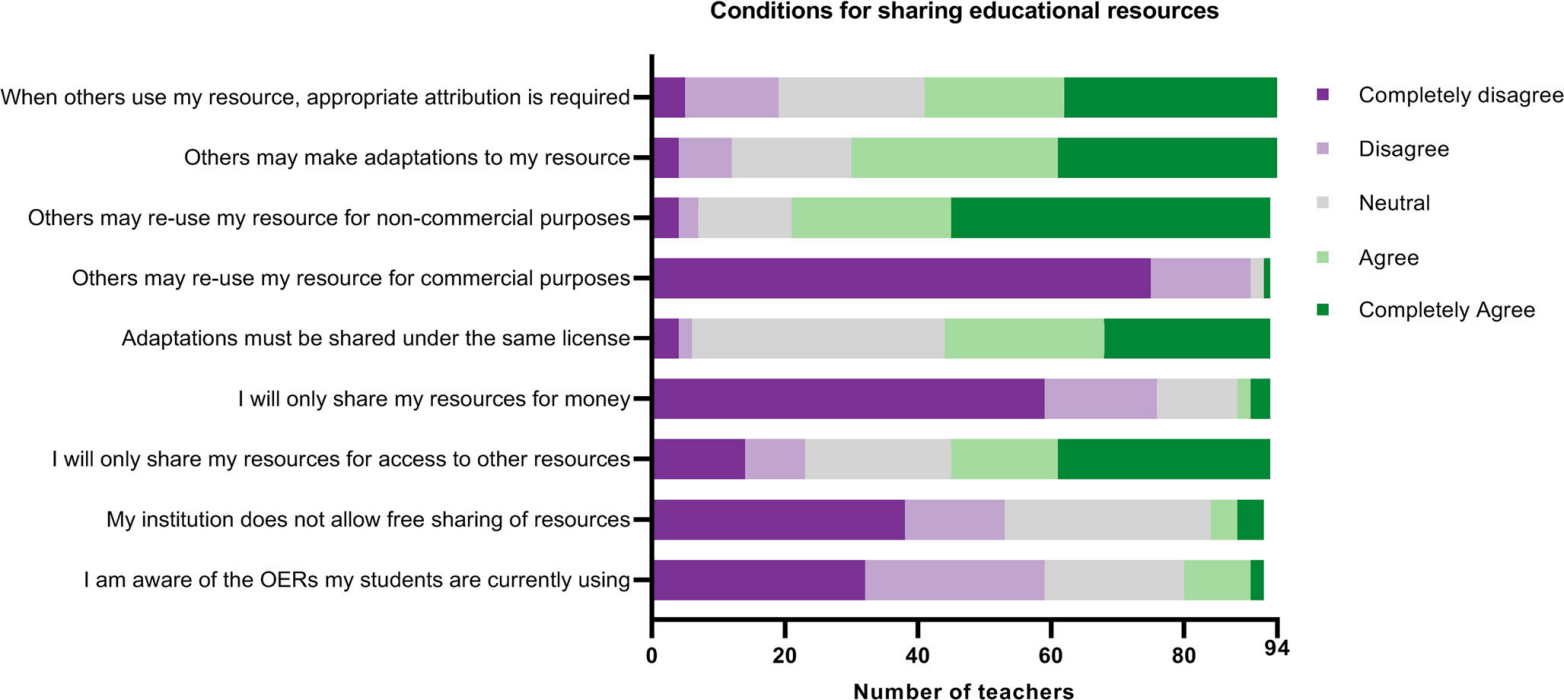
Conditions under which teachers are willing to share their resources. Statements 1 through 5 are lay descriptions of Creative Commons Rights, on which Table 1 is based. *OERs* open educational resources

Ninety-four teachers rated the conditions under which they would be willing to share their resources (Fig. 2). Only 8.7% thought their institution had a policy that prevented the sharing of resources, and only 13% knew what open resources their students used. The copyright licenses that teachers felt most comfortable with were based on the first five statements and shown in Table 1. Only two teachers chose licenses that would allow commercial use (1 CC BY and 1 CC BY-SA); 86% of the teachers chose an “open” license (i.e. one that allows adaptations).

### Opportunities and challenges for the open educational resource platform

The teachers could indicate opportunities and challenges to the use of the online open educational resources platform. Thematic analyses revealed a total of 7 opportunities in 20 subthemes and 7 challenges in 15 subthemes (Table 2). Some quotes to exemplify the advantages include the following:

**Table 2:** Opportunities and challenges of the online open educational resource and collaboration platform

Opportunities		Challenges	
**Theme -** subtheme	**n**	**Theme -** subtheme	**n**
**To get access to resources**	**25**	**Language barriers**	**29**
… of high quality	3	**Local differences**	**21**
… more up to date	3	… in prescribing ethics, guidelines and drug availability	10
… in a greater variety	3	… in curricula/teaching methods	11
… for free	2	**Investment of time and money**	**21**
**To get inspired**	**18**	… for maintenance of the platform	8
… on new teaching approaches	5	… difficulties obtaining funding	4
… and adjust own content accordingly	2	… to find and adapt the resources	4
**To improve quality**	**26**	... costs for users	**5**
… via specialized resources	2	**Technological barriers**	
… by comparing to others	5	… the need for a very clear overview and indexation	7
… via peer-reviewed resources	2	… compatibility issues with currently used software	2
**To harmonize curricula, learning goals, and teaching**	**18**	…. limited availability of on-campus computers	1
… in Europe	6	… digital resources do not fit in the current curriculum	1
**To contribute**	**14**	**Quality control**	**10**
**To improve collaboration**	**14**	… risk of low-quality content	**7**
… by learning from local differences	5	… the requirement for quality control	3
… by promoting harmonization	4	**Problems with ownership**	**5**
… by scientifically evaluating educational resources	3	… copyright issues	3
… by communicating about meetings and conferences	2	… a lack of responsibility for what is placed on the platform	1
**To save valuable resources**	**9**	… poor return value for sharing	1
Time	6	**Dissemination problems**	**2**
Time and costs	3	**No barriers**	**10**
**Unthematized codes**		… if cost-free	3
Because it is easy	5	Undefined codes*	**4**
To assure sustainability	1		
To fulfil the internationalization policies of my university	1		
Due to the possibility to link w/ national drug databases	1		
**Codes with reservations rather than advantages**			
Only if it is aligned with my teaching/regional situation	3		
Only if it is better than currently available	2		
Only teachers should have access	1		
What’s the point, similar project exists	1		

Themes in bold; corresponding subthemes underneath. Subtheme numbers do not add up to theme total, because not every code was given a subtheme. *The following codes were not thematised because they were insufficiently understood: “Need for blended learning”, “Need to define learning goals”, “Depends on topics and subjects”, and “No practical information enough”

“To benefit my students by enabling them to access material from other institutions without having to replicate it ourselves”. (theme: To access resources)

“Finding new ideas to explain CPT”. (theme: To get inspired, subtheme: on new teaching approaches)

“I think that using this platform could improve the level of education, as I would be able to compare my way of teaching students with that of other CPT educators”. (theme: To improve quality of teaching and resources, subtheme: by comparing to others)

“To standardize the requirements among native and foreign students, to find what is common to European pharmacology education”. (theme: to harmonize teaching, subtheme: in Europe)

The following quotations illustrate some of the challenges:

“One barrier could be language. Teaching in most countries will probably not be in English but rather in the national language. Therefore, many resources either must be translated or can be used only by a few native (English) speakers”. (theme: language barriers)

“Education in clinical pharmacology varies from country to country in Europe. Some topics are not present in the curricula of all countries and thus resources on such topics cannot be used efficiently by all educators”. (theme: local differences, subtheme: in curricula)

“Time to go through it, it would have to be very user-friendly to allow easy searching for fit-for-purpose learning resources”. (theme: investment of time and resources, subtheme: time required to find and adapt resources)

“Risk of garbage in, garbage out”. (theme: quality issues, subtheme: risk of low-quality content)

### Consensus on the framework of the open educational resource platform

Forty-seven conference attendees participated in the live consensus meeting in Stockholm, Sweden. Based on the aforementioned opportunities and challenges, the researchers presented the following mission statement: *We should facilitate an active network of CPT teachers to improve and harmonize teaching via shared resources, collaboration, and inspiration via means of an online platform*. After a brief discussion, it was emphasized that CPT teachers include graduate and undergraduate pharmacology and clinical pharmacology teachers of any background. No other alterations were proposed, and the mission statement was accepted.

Next, the possible solutions for local differences were discussed. The main local differences were prescribing ethics, local availability of medicines, differences in guidelines, and differences in curricula and educational systems. One suggestion was to provide information about these differences on the platform. Another was that the resources on the platform should mainly concern universal teaching objectives such as pharmacokinetics and medication safety. All attendees agreed that both of these solutions were fitting and easily implementable.

The easiest solution to potential language barriers would be to make the platform and its content in English. However, it was pointed out that teachers are unlikely to go through the trouble of translating their educational resources prior to uploading them onto the platform. Therefore, it was decided that the main language would be English, but that resources in other languages would be allowed. There was agreement that the platform should be free. Some suggestions for funding the platform were discussed, although none in detail.

With regard to quality management, two suggestions were made. First, users could review and rate resources. Secondly, each resource could be reviewed and validated by international colleagues. Both these suggestions were approved. Additionally, it was suggested that people who upload resources should be asked to check that the content is still up to date. However, there was no agreement about how this should be achieved, and so this aspect would be discussed in the future. In order to increase the resources available, one attendee suggested that teachers could only download resources if they had also contributed a resource, but this was perceived as being contrary to the goal of the platform. The meeting closed with a discussion of the most appropriate copyright license, based on survey findings. It was agreed that the most appropriate license would be CC BY-NC, with or without a share-alike (SA) feature. More open licenses would be welcomed, but less-open licenses (i.e. that do not allow adaptation) would greatly reduce the usability and usefulness of resources.

## Discussion

This article provides valuable insights into the attitude of teachers towards digital and open educational resources, as well as the challenges they foresee about sharing their resources on an international online platform. More importantly, this study also provides practical solutions to these challenges that can be easily implemented within the framework of such open educational resource platforms.

Several studies have identified the challenges or barriers associated with wider adoption of open educational resources [8, 10, 11]. Findings are very similar, regardless of whether they come from a large international survey amongst higher and adult education professionals [11], an action research amongst users of the Open Discovery Space for primary and secondary education [8], or a survey among teachers of physiology [10]. The OPAL report [11] categorized these challenges into five dimensions: lack of institutional support for the creation and use of open educational resources; lack of technological tools, such as insufficient computer availability; lack of skills and time to find or create open educational resources; lack of quality or fitness of open educational resources; and personal issues. With a few exceptions, our results reflect these findings. Although the lack of institutional support was not specifically mentioned, this may underlie the time (and financial)-related challenges reported. Even though the OPAL report stems from 2011 and there have been technological advances since then, technological issues still remain an important challenge, but the problems appear to have shifted from a lack of computers and internet connectivity to problems with software compatibility. One way to increase compatibility and adaptability is to specifically allow resource sharing in the intellectual property license and to provide an editable source file [17] (e.g. MS Office rather than a PDF). The lack of time and skills to find and adapt open educational resources was reported in earlier studies and remains a problem. Both in the present study and a survey of physiology teachers[10], finding suitable open educational resources on existing platforms was described as a very tedious task, much like finding a needle (suitable resource) in a haystack (of unfitting and poor-quality resources). This is directly related to another dimension of the OPAL study: a lack of quality or fitness. Language barriers, local differences, and poor quality all limit the applicability of a resource and were the issues that most concerned the teachers in our study. Several promising solutions were suggested in the consensus meeting. In addition, we believe that the dedicated nature (CPT education) of the proposed platform will prevent it from becoming overwhelming or confusing. Moreover, limiting access to the platform to teachers only will improve the quality of its content, because this allows curation, adaptation to the local standards, and (if required) translation before content is used for teaching. The last dimension (called “personal issues” in the OPAL report) basically describes the “not invented here syndrome”, which is a distrust of products created elsewhere [18]. This was not specifically mentioned in the present survey, but probably underlies some teachers’ concerns about the quality of resources. We believe that the past accomplishments of the EACPT’s education working group, close collaborations in the Network Of Teachers In Pharmacotherapy, and well-established partner universities will help to reduce this distrust. Not mentioned in the OPAL report, but reported as a challenge by our teachers and in other studies, is the question of who is responsible for the correctness and currency of the resource after distribution? [8] The main consensus is that it is always the user and never the original author who is responsible. However, to avoid inaccuracies in later adaptions which might reflect badly on the original author (and his/her institution), it is important to work with alternate versions that coexist alongside (and not replace) the original resource.

**Fig. 3:**
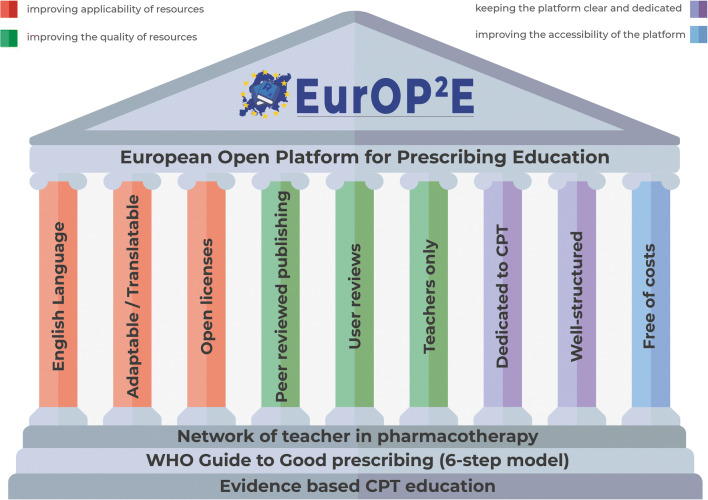
The framework for the European Open Platform for Prescribing Education (EurOP^2^E). *CPT* clinical pharmacology and therapeutics

Based on the survey, consensus meeting, and previous literature, we created the following framework for the platform (Fig. 3). It should be built on a strong foundation of evidence-based CPT education, problem-based learning according to the WHO Guide to Good Prescribing, and the active NOTIP community. The pillars represent the essential aspects that will be incorporated in the platform. To maximize the user-friendliness of the platform, it will be kept small, dedicated to CPT, and clearly organized according to previously established key learning outcomes. In order to improve resource applicability, the content will be primarily in English, easily adaptable and translatable, and licensed in a way that allows re-use, adaptation, and redistribution at no cost. To ensure resource quality, resources will be peer-reviewed both prior to publication and after publication (by means of a star-rating and user comment-system). Moreover, limiting platform access to teachers will help ensure resource quality because teachers can curate and adapt resources before using them in teaching. While these measures may provide practical solutions for other open educational resource platforms as well, it must be stressed that ours is not a conventional open educational resource repository. One may even wonder how “open” our platform really is, if we choose to restrict access to teachers only. Our goal is not to facilitate self-learning, but rather to improve existing institutional CPT education. Therefore, we aim to help CPT teachers (in Europe and elsewhere) to make their education more problem-based and provide them with tools to do so. The exact nature of these tools is to be decided in the peer review process and may vary from complete courses to showcases of best practices and teach-the-teacher trainings. In analogy to the simultaneous European Prescribing Exam project (EuroPE^+^), this project was named EurOP^2^E: European Open Platform for Prescribing Education (www.prescribingeducation.eu).

Recently, due to the COVID19 pandemic, teachers worldwide got faced with the challenge to transfer their onsite education to online education. In addition to the aforementioned goals, EurOP^2^E will help to assure that we can continue to train medical and non-medical prescribing students to safely prescribe medicines during these challenging times.

Several limitations to our study must be acknowledged. First, as with any uncontrolled study with voluntary participation, the results of both the survey and consensus meeting may have been biased. Teachers who are inherently interested in open and digital education may have been more likely to participate than others, thus giving a slightly one-sided view. Secondly, the survey was large and took approximately 20–30 min to complete, which may have discouraged teachers from participating and may explain why some answers were not detailed. Not all NOTIP associates had the possibility to travel to Stockholm, Sweden, to attend the conference and consensus meeting. As signing in for the consensus meeting was not obligatory, the number of attendees (*n*=47) may have been underestimated. Lastly, the consensus meeting lasted only 60 min, which means that some topics were not discussed or discussed inadequately.

## Conclusion

Teachers recognize many potential advantages of using digital and open educational resources but also the challenges to more widespread use and creation of such resources. The expected challenges mostly revolve around the applicability of resources, such as language barriers, local differences, or quality concerns, but also a lack of time and technological barriers. We aim to remove these barriers by providing a framework for a free, easy-to-use platform dedicated to CPT education for CPT teachers, with English language, peer-reviewed, universal open educational resources that are easily adaptable and translatable to accommodate local differences.

## Implications for further research

In order to make the platform successful, more needs to be learned about the educational content that international CPT teachers are currently missing and which could be collaboratively produced and made available on the platform. Moreover, future research should aim to find the criteria on which to base peer review of proposed resources.

## Supplementary information

ESM 1 (DOCX 16 kb)

## Data Availability

The data that support the findings of this study are available from the corresponding author upon reasonable request.

## Declarations

### Code availability

N/A.
