# Supplementary material for: EurOP2E – the European Open Platform for Prescribing Education, a consensus study among clinical pharmacology and therapeutics teachers
Source: Eur J Clin Pharmacol. 2021 Feb 23;77(8):1209–18. doi: 10.1007/s00228-021-03101-4 (PMC8275529; doi:10.1007/s00228-021-03101-4)
Supplement: Supplementary file 1 — (DOCX 16 kb) [file 228_2021_3101_MOESM1_ESM.docx]

**Supplementary materials

Supplementary table 1: Reasons behind current sharing practices**

| **Reasons to share / collaborate** 30 medical schools | **Reasons not to share / collaborate** 33 medical schools |
| --- | --- |
| **To use resources more efficiently (n= 9)**  “Academic funding should […] benefit the whole community”, “Because it is a waste of time and energy if people have to re-invent the wheel”, “Not to re-do something that has been elsewhere” | **No network / because nobody asked (n=9)** “Actually we did not receive any requests for collaboration”, “I was not asked”, “No network available” |
| **To increase availability (n=5)** “To cover more students”, “to be used in second chance education”, “To make it available to others” | **I do not know / would be happy to share (n=3)**  “I don’t know”, “No specific reason”, “We’ll be delighted to share with many more people” |
| **To harmonize and standardize content (n=4)**  “To harmonize the national curriculum”, “To reach an interuniversity diploma”, “To standardize teaching” | **Technical reasons (n=6)** “It is a stationary type computerized virtual patient”, “Hosted on a local platform”, “a lot of departments do not use Moodle”, “still in development” |
| **To learn from the experience of others (n=5)**  “Peer review and peer usage”, “sharing cases and experiences”, “opportunity to cooperate” | **Language issues (n=5)**  “Only available in Slovenian”, “It is in Spanish, but we’ll be happy to share anyway” |
| **To increase / warrant quality (n=3)**  “To ensure prescribing knowledge of students who graduate our university”, “peer review and peer usage”, “Every teacher is the best in something” | **Too specific / not special enough (n=7)**  “Other universities already have this”, “these resources are available online” |
| **Because this resource is meant to be shared (n=8)**  “It is a collaborative product”, “[specific resources] are used throughout the UK”, “We do not actively share, but it is freely available” | **Copyright / privacy law restrictions (n=2)** “The reason for restricting is patient-specific data”, “copyrighted material developed in conjunction with the pharmaceutical industry” |
| *Answers that contained no reasons to share: - It is a teaching resource available at our institution - This resource was created for use by healthcare professionals, it is also used in education*  *- We appreciate any resource available. We are also keen on to share our own resources if these may be of any interest.* | *Other reasons not to share:*  ***-*** *Academic independency - Personal sources - We do not have our own CPT resources to share* |

**Supplementary table 2: The advantages and disadvantages of digital educational resources**

| **Advantage** | **Times answered** | **Disadvantage** | **Times answered** |
| --- | --- | --- | --- |
| **Re-usable** | 73 | **Creation costs much time** | 79 |
| **Preferred by students** | 58 | **Creation costs much money** | 65 |
| **Easy to share with other universities** | 54 | **Lack of technical skills** | 30 |
| **Easy student activity tracking** | 50 | **Less effective** | 6 |
| **Multimedia** | 42 | **Other disadvantages**   - Students learn questions instead of questioning themselves about a patient - No personal contact, which is important for doctors - Digital education should have a proper didactical design - Copyright issues, privacy of patient data - Only pre-specified non-individual feedback - Maintenance of program in ever changing ICT environment (browsers- PC systems- University network systems - Dependent on other clinicians to contribute content - Lack of evidence that it is more effective - Far less human interaction - less personal contact to students - When offered the choice, students rather used the simple than audio-recorded PowerPoint presentations - It is managed by BPS UK so we have no access to questions actually asked - If made available in open access, any evaluation becomes quickly obsolete, and less effective, since students learn the minimum necessary to answer already known questions and do not go through the whole subject. - Lack of cooperation between universities, but also lack of cooperation between universities and health care system. - Uptake by very small proportion of students - Speeds up the progressive loss of contact between teachers and students. - Rather ineffective as stand-alone educational tool | 18 |
| **Cost efficient in creation** | 27 |  |  |
| **More effective** | 27 |  |  |
| **Promoted by institution** | 20 |  |  |
| **Other advantages**   - Students can study at their own tempo, at the place they like most - We prepare student for a global environment - No "wet " lab activities (bio-assays) - Instant Feedback - complex relationships can be explained more easily- students can use it repeatedly until they achieve the objective - Always up to date - Can be used when students are in remote hospitals - It aligns with the development in health care, where eLearning, CDSS, electronic prescribing and electronic health care systems, including the use of quality registers are part of daily work routine - Students can go over it multiple times and ask further questions - Caters to changing learning attitudes. Allows for interactivity, simulating something of a real-time application. - Compulsory preparation of students before further educational sessions | 11 |  |  |
